# Automatic Identification of Interictal Epileptiform Discharges in Secondary Generalized Epilepsy

**DOI:** 10.1155/2016/8701973

**Published:** 2016-06-09

**Authors:** Won-Du Chang, Ho-Seung Cha, Chany Lee, Hoon-Chul Kang, Chang-Hwan Im

**Affiliations:** ^1^Department of Biomedical Engineering, Hanyang University, Seoul 04763, Republic of Korea; ^2^Department of Pediatrics, Severance Children's Hospital, Epilepsy Research Institute, Yonsei University College of Medicine, Seoul 03722, Republic of Korea

## Abstract

Ictal epileptiform discharges (EDs) are characteristic signal patterns of scalp electroencephalogram (EEG) or intracranial EEG (iEEG) recorded from patients with epilepsy, which assist with the diagnosis and characterization of various types of epilepsy. The EEG signal, however, is often recorded from patients with epilepsy for a long period of time, and thus detection and identification of EDs have been a burden on medical doctors. This paper proposes a new method for automatic identification of two types of EDs, repeated sharp-waves (sharps), and runs of sharp-and-slow-waves (SSWs), which helps to pinpoint epileptogenic foci in secondary generalized epilepsy such as Lennox-Gastaut syndrome (LGS). In the experiments with iEEG data acquired from a patient with LGS, our proposed method detected EDs with an accuracy of 93.76% and classified three different signal patterns with a mean classification accuracy of 87.69%, which was significantly higher than that of a conventional wavelet-based method. Our study shows that it is possible to successfully detect and discriminate sharps and SSWs from background EEG activity using our proposed method.

## 1. Introduction

Epileptiform discharges (EDs) are signal patterns frequently observed in interictal electroencephalogram (EEG) of patients with epilepsy [1]. EDs are generally utilized by expert epileptologists to assist in the diagnosis of epilepsy [2]. However, manually labeling various types of EDs is time-consuming and labor-intensive because EEG data are generally recorded from many electrodes during continuous monitoring of patients.

For this reason, the automated detection of EDs has drawn researchers' attention. Automatic detection algorithms introduced in the literature mostly focus on either sharp-wave (sharp, sometimes referred to as spike) or sharp-and-slow-wave (SSW) discharges. Epileptiform sharps are detected using a variety of algorithms such as deterministic finite automata (DFA) [3], geometrical features and artificial neural networks [4], cross-correlation [5], and dynamic time warping [6]. A recent review reported that these algorithms can detect epileptiform sharps with approximately 90% accuracy [7]. Algorithms for the automatic detection of SSWs have also been introduced in the literature. Although the number of studies to design algorithms for SSW detection is relatively less than that for sharps detection, a variety of approaches have been used for the automatic detection of SSWs, such as Fourier transforms [8], wavelet transforms [9–11], Gotman and Gloor's spike detection method [12], a rule-based system [13], and a detection method using a number of geometrical features [14]. It was also shown that sharp-and-wave discharges can be distinguished from sleep spindles and background activities using wavelet-based methods [9, 15–18].

There have been few studies to detect different types of EDs together without identifying their individual types. D'Attellis et al. detected EDs including spikes and spike-wave complexes using a wavelet transform [19]. They also detected three types of EDs (spike, slow-wave, and spike-and-slow-wave) simultaneously by using wavelet transform coupled with a multilayer perceptron model [20]. Bergstrom et al. proposed a method utilizing total wavelet decomposition and signal variation to identify four types of EEG segments: normal, seizure, spike, and other abnormal types [21]. They successfully distinguished spike and seizure segments from background activity on EEG; however, the accuracy for identifying other abnormal types was relatively low (approximately 44% were classified as a normal segment).

In this study, we propose a new method based on a novel wave detection algorithm for the detection and identification of different types of EDs. The proposed method was evaluated with intracranial EEG (iEEG) data recorded from a patient with Lennox-Gastaut syndrome (LGS), a type of secondary generalized epilepsy in which the patient suffers from multiple seizure types, therefore generating a variety of EDs on iEEG [22]. LGS is known to be treatment resistant, but recent studies have shown that surgical treatment is possible for some patients with LGS that have focal epileptogenic zones [23]. However, because of their generalized ictal iEEG discharges, surgical resection areas are determined mostly based upon interictal iEEG patterns and functional neuroimaging results [23].

Although the automatic identification of interictal EDs is of great importance for surgical planning in patients with LGS, no studies have attempted to develop algorithms for detecting and identifying interictal EDs. Among the various interictal EDs, we have focused on repeated sharps and runs of SSWs, as these forms are hard to detect with existing algorithms. These characteristic EEG patterns have great potential to aid in surgical planning of patients with secondary generalized epilepsy. The performance of our proposed algorithm was evaluated and compared with that of conventional frequency domain approaches.

## 2. Materials and Method

### 2.1. Materials

**Figure 1:**
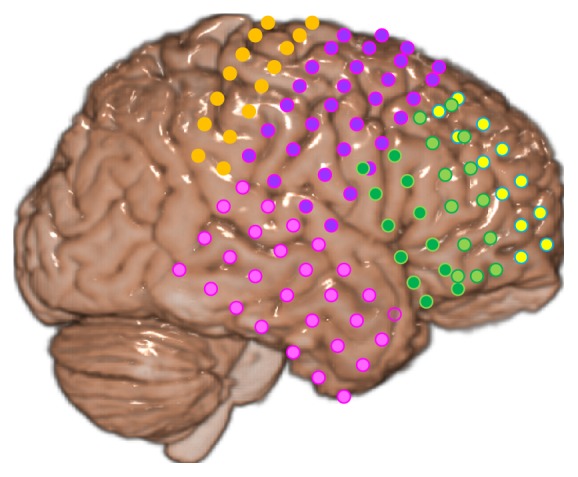
Electrode placement: six different electrode grids are depicted with different colors.

We used iEEG data recorded from a pediatric patient with LGS (17-year-old male) at Severance Children's Hospital. The data was recorded at a sampling rate of 1600 Hz with a band-pass filter at 1–500 Hz. A 60 Hz notch filter was also applied before the analog-to-digital conversion. As shown in Figure 1, 128 grid electrodes were placed over the cerebral cortex of the right hemisphere to cover the prefrontal, frontal, and temporal lobes.

**Figure 2:**
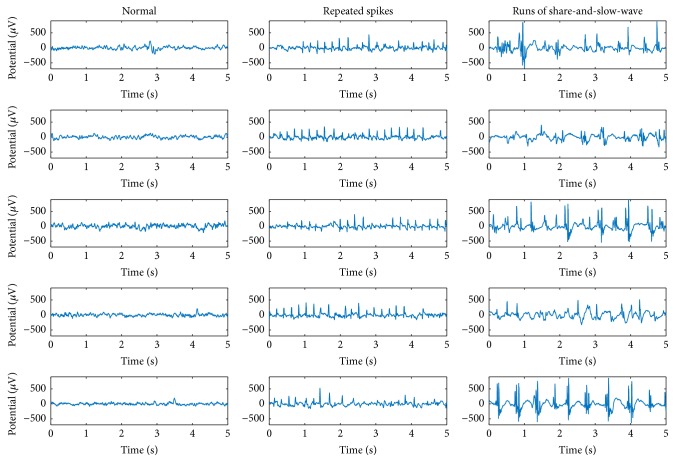
Examples of segments with epileptiform discharges and normal segments.

A medical doctor manually labeled the types of EDs and a biomedical scientist who was not involved in developing the algorithm in this study adjusted the ranges. We extracted five-second epochs including repeated sharps and runs of SSWs from the background activity. We identified 73 segments of repeated sharps, 1,752 segments of SSWs, and 91,701 normal segments without any specific EDs (Figure 2).

### 2.2. Wavelet Features

Wavelet transform is one of the most popular approaches for the analysis of EDs [9–11, 19, 20]. In this study, we adopted techniques introduced by Übeyli et al. which were simple to use while providing promising results [10]. The features used were mean, maximum, minimum, and standard deviation of the wavelet coefficients in each scale. A wavelet coefficient *C*_*b*_^*a*^(*s*) of data *s* at scale *a* and position *b* is defined as

(1) $$\begin{array}{c} C_{b}^{a}\left(\;s\;\right)=\overset{\infty }{\underset{-\infty }{\int }}s\left(\;t\;\right)\frac{1}{\sqrt{a}}\bar{\psi \left(\frac{t-b}{a}\right)}dt, \end{array}$$

where *ψ* represents a Morlet mother wavelet. The number of scales was empirically set to 10 in this study.

### 2.3. Detection of Sharps and SWs

We have developed an algorithm for detecting sharps and SWs. Although there is a conventional method for the detection of SSWs [14], it assumed that a sharp always accompanies a SW. Though this assumption is useful in distinguishing SSWs from normal EEG data, it is not appropriate for classifying repeated sharps and runs of SSWs because both types of EDs commonly include sharps.

**Figure 3:**
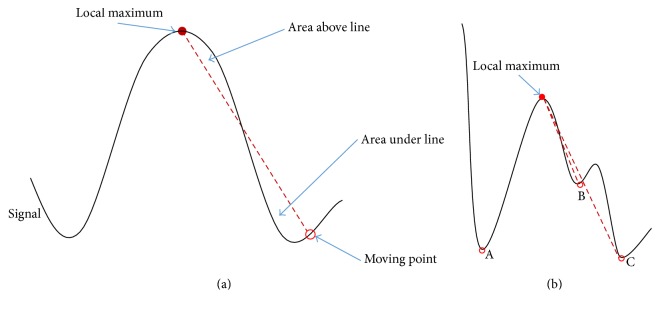
Schematic illustrations to elucidate the wave detection algorithm: (a) depiction of the AAL and AUL; (b) A, B, and C denote the candidates of the wave boundary, when more than one boundary-point candidate is found for a local maximum.

We designed an algorithm that distinguishes sharps and SWs based on the sharpness of the waveform pattern (Figure 3). To determine the range of a wave that can potentially be either sharp or a slow-wave, a line is drawn from a local maximum to a moving point (MP) near the maximum. Then, as shown in Figure 3(a), two areas can be calculated for each MP, which are (1) area under the signal and above the line (denoted by AAL), and (2) area above the signal and under the line (denoted by AUL). The AAL and AUL from a local maximum to an MP are calculated with the following equations:

(2) AALx,y=∑i=xysi−li−x+1 ∣ si−li−x+1>0NAULx,y=∑i=xyli−x+1−si ∣ si−li−x+1<0NN=sx−sy+y−x+1lk=sy+k−1·sx−syx−y,

where {*f*∣*C*} denotes a set in which elements are generated by a function *f* while a condition *C* is satisfied, ∑{·} denotes the summation of all the elements in a set, *x* and *y* denote the data indices of the local maximum and MP, respectively, *s*_*i*_ is the *i*th data point of the signal, and *N* is a normalization factor. The purpose of AUL is to find candidates for two boundary points (leftmost and rightmost points) of a wave. To search for the candidates of the current wave under consideration, the MP is gradually moved outward (to the left or to the right) from the local maximum. A point is determined to be a candidate of the wave boundary if the AUL at the point is smaller than the preset threshold (*θ*_AUL_) and the AUL at the next point is greater than or equal to the threshold. After finding all candidates for a local maximum, all possible pairs of preceding and succeeding candidate points (one from the candidate points preceding the local maximum and the other from the candidate points succeeding the local maximum) are tested to identify the wave types (e.g., in Figure 3(b), two pairs A-B and A-C are tested). A wave between two candidate points is classified as a sharp if AALs of both points are smaller than the threshold (*θ*_AAL_), and their amplitudes are greater than or equal to the threshold (*θ*_amp_). The pattern is classified as a SW if the AAL of a candidate point is bigger than the threshold (*θ*_AAL_) and the amplitude is greater than or equal to the threshold (*θ*_amp_). In addition, any sharp or SW with length (duration) outside expected range of an ED is discarded since combining two candidates may indicate a waveform with longer duration than a sharp. Waveforms with unbalanced ascending and descending length are discarded as well. For example, a wave is discarded if bal_amp < *θ*_bal_amp_, where bal_amp = *A*_small_/(*A*_small_ + *A*_big_), *A* denotes amplitude of ascending or descending slope, *A*_small_ denotes the smaller amplitude, and *A*_big_ denotes the bigger amplitude. We call bal_amp the “balance in amplitude.” The “balance in time,” which is used for further processing, can be calculated in a similar manner: bal_time = *L*_short_/(*L*_short_ + *L*_long_), where *L*_short_ denotes the shorter time length and *L*_long_ denotes the bigger length. After testing for all possible pairs, all the overlapping ranges found for a local maximum were merged into a single range, which was then used as a feature for the automated classification of EDs.

In later experiments, we used different amplitude thresholds for the sharps and SWs because the amplitudes of sharps and SWs generally differ from each other. Moreover, the moving range of MP was restricted between [*R*_*s*_, *R*_*e*_], where both *R*_*s*_ and *R*_*e*_ are the distances from the local maximum under consideration.

The wave detection algorithm can be summarized as follows:

(1) Find all the local maxima of the signal, which are candidates of a wave peak.
(2) For each local maximum point, the following steps are conducted:
  (A) For each direction (left or right),
    (i) set an MP at *R*_*s*_ away from the local maximum. Let the number of MPs be denoted as *i*,
    (ii) draw a line from the local maximum to the MP,
    (iii) calculate AUL,
    (iv) set MP_*i*−1_ as a candidate of the wave-end if AUL_*i*−1_ < *θ*_AUL_ and AUL_*i*_ ≥ *θ*_AUL_,
    (v) move the MP a step outward,
    (vi) repeat steps (ii) to (v) until the distance of MP from the maximum reaches *R*_*e*_.
  (B) Check for all possible pairs of candidates if they satisfy conditions of sharps or SWs and merge all the (overlapping) ranges of the satisfied pairs.

### 2.4. Procedure of the Proposed Method (Figure 4)

The wave detection algorithm in Section 2.3 was designed to detect waves whose deflections are downward. Since inverting signals would be inconvenient to satisfy this condition, we introduce a simple automatic procedure for a signal to be set upright (the deflection of waves is downward). This procedure is conducted for each data segment independently. First, the sharps are detected from both the original and inverted signals, and then we determined which signal is upright by evaluating the “dominance” of the detected sharps. The dominance of a signal is calculated using the following equation:

(3) $$\begin{array}{c} V=\overset{N}{\underset{i=1}{\sum }}\min\left\{\;L_{i}^{ascend},L_{i}^{descend}\;\right\}, \end{array}$$

where *N* is the number of detected sharps and *L*_*i*_^ascend^ and *L*_*i*_^descend^ denote the lengths (duration) of ascending and descending blocks of the *i*th sharp. The signal with the larger *V* value is deemed to be the upright signal. Please note that the channel information of the segment is hidden to the wave detection algorithm and the sharps in a segment are detected when determining the dominance. Afterwards, the SWs were detected from the upright signal.

The parameters for the detection of sharps and SWs were derived from a sample dataset, which was randomly selected from all the data segments. Ten segments were selected from each type of ED and the ranges of EDs were labeled manually. Geometrical features such as AUL and AAL were calculated for each of the EDs, and the parameters were determined based on the mean and standard deviation to include most EDs in the sample dataset, as follows:

(4) θAULtype=AULtype−+σAULtypeθAALsharp=AALsharp−+σAALsharpθAALSW=AALSW−+σAALSWθamptype=amptype−−σamptypeθbal_amptype=bal_amptype−−σbal_amptypeθbal_timetype=bal_timetype−−σbal_timetypeRs=θbal_timetype·Wtype−−σWtypeRe=1−θbal_timetype·Wtype−+σWtypeERtype=Wtype−−σWtype,Wtype−+σWtype,

where the subscript type represents either sharp or SW, ER_type_ means the expected range of an ED, AUL/AAL_type_, amp_type_, bal_type_, and *W*_type_ denote AUL/AAL, amplitude, balance, and wave-width of an ED, respectively. The operator $\overset{-}{\cdot }$ denotes mean and *σ* denotes standard deviation. Please note adding or subtracting standard deviation depends on the types of thresholds. The standard deviation is added when waveforms with geometrical feature values bigger than a threshold are to be discarded and is subtracted if waveforms with the values smaller than the threshold are to be discarded. The derived parameters are shown in Table 1.

The next step for identifying EDs is calculating geometrical and relational features from the source signal and the detected waveforms. Total 27 features were used in this study (Table 2), in which most features except *f*2, *f*14, *f*26, and *f*27 were introduced in the study of Olejarczyk et al. [14]. Some features were used as they were in the literature, while others were slightly modified. For example, an original feature “ratio of duration of a wave's increasing part to the amplitude” was changed to “ratio of duration of a wave to the amplitude.” The new features, *f*2 and *f*14, were introduced to check the presence of suspected EDs and *f*26 and *f*27 were introduced to describe detailed characteristics of SSWs because sharps and SWs are often accompanied by each other.

Lastly, the support vector machine (SVM) was used for the classification of EDs. A Gaussian radial basis function was empirically chosen for a kernel. To classify three different types of segments using SVM, a two-step classification approach was employed (denoted by three-class classification in Results). This approach was used to distinguish between normal signals and EDs and subsequently between repeated sharps and SSWs. We also tested two-class classifications for each pair of segment types (denoted by pairwise classification in Results). We used a statistics and machine learning toolbox implemented in Matlab (ver. 10.1, MathWorks, USA). The parameters used for SVM are listed in Table 3.

The proposed method was evaluated using 10-fold cross-validation. The segmented iEEG data were shuffled and divided into ten groups so that each group has the same number of EDs and normal segments. SVM was trained using data from nine groups and then the remaining data were tested. The training and testing were repeated ten times so that all the data were included. To avoid any failures in training caused by severely unbalanced data, only 20,000 randomly selected data points were used for training normal signal patterns.

For validation of the proposed method, the wavelet transform introduced in Section 2.2 was also used for the classification with SVM. After the 10-fold validation was finished, the accuracy of each method was averaged over all validation iteration. The accuracy of a method was calculated as follows for each iteration:

(5) Acc=∑i=1CSensi+Speci2CSensi=TPiTPi+FNi,Speci=TNiTNi+FPi,Seli=∑i=1CTPi∑i=1CTNi+FPi,

where *C* denotes the number of target classes, Sens_*i*_ denotes the sensitivity of a target class, Spec_*i*_ denotes the specificity of the class, Sel_*i*_ denotes the selectivity [24] of the class, TP_*i*_ denotes the number of correctly classified segments for the class, and FN_*i*_ denotes the number of target segments that were not classified correctly. We used the mean of the sensitivities for all the classes instead of the general definition of the accuracy because the numbers of segments were severely unbalanced in our data. Statistical significance was tested using Wilcoxon's signed rank test.

## 3. Results and Discussion

### 3.1. Binary (Pairwise) Classification

Table 4 lists the classification accuracies of the methods used for the distinction between segments with EDs and normal patterns. As shown in the table, the proposed method could stably detect the EDs from normal segments. The average accuracy of the proposed method was 93.76%, which is 2.51%*p* higher (Bonferroni corrected *p* = 0.006) than the conventional wavelet-based method (average accuracy = 91.25%). The accuracy was significantly decreased (91.96%) when the wavelet features were utilized together with the proposed method (Bonferroni corrected *p* = 0.0117). One of the main reasons for this decrease could be that the number of features was increased dramatically to 67 features, whereas a decrease could have been overcome by reducing dimensions or selecting optimal feature sets [25]. The selectivity was increased when the wavelet features were utilized together with the proposed method. This is because that the sensitivity and specificity were unbalanced and the number of normal segments was relatively bigger than ED segments.

Table 5 lists the accuracy of classification between repeated sharps and SSW segments. The accuracies were generally lower compared to the previous binary classification between EDs and normal segments. In particular, the sensitivities of the wavelet-based method were severely inconsistent over iteration, reporting a number of extremely low sensitivities. The mean sensitivity for the sharp segments barely reached 57.14%. Although the mean sensitivity and selectivity for the SSW segments exceeded 90%, it was caused by the low sensitivities of the sharps identification.

In contrast to the wavelet-based method, our proposed method kept the sensitivities balanced in both classes. The mean sensitivities for the repeated sharp and SSW segments over iteration were 79.76% and 73.99%.

### 3.2. Three-Class Classification

Table 6 lists the accuracies of the classification of three different types of iEEG segments: normal, repeated sharps, and SSW. We used the same parameters as the previous pairwise tests. The overall accuracy of our proposed method was 87.69%, which was significantly greater than that of the wavelet-based method (81.97/86.96%, *p* = 0.0176). The mean accuracy of the combined method (utilizing proposed and wavelet features together) was slightly lower (0.74%*p*) than that of the proposed method, although the difference was statistically insignificant. The sensitivities of each class trended similar to those of the previous pairwise tests. Since the classifier for the three classes was composed of binary classifiers, the mean sensitivities for the normal class were similar to those in Table 4. The slight difference (less than 0.1%*p*) originated from the random selection of normal segments in each iteration number. The low accuracy of the wavelet-based method was mainly caused by a failure in identifying sharps. The sensitivity of the wavelet-based method for identifying repeated sharps was 32.86 ± 16.14%, which was dramatically lower than the previous binary classification between repeated sharps and SSWs (57.14%  ±  28.10%), implying that the detection of sharps was less successful than that of SSWs. In contrast, our proposed method demonstrated well balanced sensitivities in detecting repeated sharps and SSWs, as in the previous binary classification results (Table 5).  Table 7 lists the distribution of outputs for each type of iEEG segments. Repeated sharp segments were misclassified as normal segments more frequently than the SSW segments when the wavelet-based method was used. In contrast, the confusion rates of repeated sharps and SSWs into normal segments were relatively similar to each other, when the proposed method was utilized solely or together with the wavelet features.

**Figure 5:**
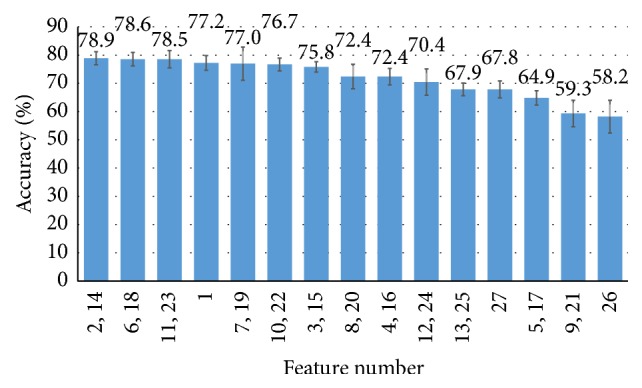
Accuracy of three-class classification when each feature group was independently used for the classification.

To evaluate the relative importance of the features used, we evaluated accuracies of each feature group (Figure 5). The best feature group in this test was the number of detected waveforms (2–14), which resulted in a classification accuracy of 78.9%. The second and third feature groups (6–18 and 11–23) also showed high classification accuracies similar to the accuracy of the first feature group. The second-best group was the “mean ratio of amplitude to wave-width of detected waveforms” and the third-best group was the “difference between the maximum and minimum amplitude of undetected blocks,” which showed 78.6% and 78.5% classification accuracies, respectively.

A limitation of this work would be that the proposed method was tested only for a single patient's data, which makes it difficult to claim that the proposed method is better than a wavelet-based method. Instead, we would like to emphasize that we developed a new method to classify two different types of EDs and background activities. Since the proposed method achieved fairly high classification results with the parameters determined from 20 randomly selected segments only, we expect that the same approach would work for other patients' data if the shapes of EDs are stable.

## 4. Conclusion

In this paper, we have proposed a novel method to distinguish two types of EDs, repeated sharps and runs of SSWs, from normal iEEG background activity in a patient with LGS. Our proposed method was developed to detect sharps and SWs independently and to classify the categories based on the geometrical and relational features calculated from the detected sharps and SWs. The results showed that we can differentiate EDs with fairly high accuracy (87.69 ± 4.11%), which is greater than a conventional frequency-analysis-based approach (accuracy of 81.97 ± 2.24%). Our method can be used as an auxiliary tool for the surgical planning of intractable epilepsy such as LGS.

## Figures and Tables

**Figure 4:**
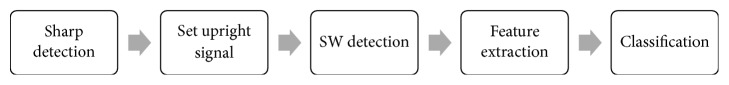
Flowchart used to describe our method.

**Table 1:** Parameters derived in the experiments.

Target waveform	Parameters	Value
Sharp	*θ*_AUL_	6.21
	*θ*_AAL_	2.92
	*θ*_amp_	148.21 (*μ*V)
	*θ*_bal_amp_	0.34
	*θ*_bal_time_	0.33
	*R*_*s*_	14.00 (ms)
	*R*_*e*_	54.77 (ms)

Slow-wave	*θ*_AUL_	5.95
	*θ*_AAL_	0.85
	*θ*_amp_	82.26 (*μ*V)
	*θ*_bal_amp_	0.34
	*θ*_bal_time_	0.26
	*R*_*s*_	29.81 (ms)
	*R*_*e*_	235.21 (ms)

**Table 2:** A full list of features used for the classification of ED segments: *S*_ALL_ denotes all data (source signal) in a segment; *S*_NS_ denotes data in the nondetected (as a sharp or SW) block only within the segment. Some features are paired (e.g., numbers 9 and 21), which can be calculated for sharps and SWs independently.

Feature number	Description
1	Standard deviation of amplitude for *S*_ALL_

2, 14	Number of detected waveforms

3, 15	Median wave-width of detected waveforms^*∗*^

4, 16	Median amplitude of detected waveforms

5, 17	Mean kurtosis of detected waveforms^*∗*^

6, 18	Mean ratio of amplitude to wave-width of detected waveforms^*∗*^

7, 19	Standard deviation of amplitude for *S*_NS_

8, 20	Feature 8 = feature 1/feature 7 Feature 20 = feature 1/feature 19

9, 21	Feature 9 = feature 4/feature 7 Feature 21 = feature 16/feature 19

10, 22	Max(*S*_ALL_) − min(*S*_ALL_)

11, 23	Max(*S*_NS_) − min(*S*_NS_)

12, 24	Feature 12 = feature 4/feature 11 Feature 24 = feature 16/feature 23

13, 25	Mean skewness of detected waveforms^*∗*^

26	Mean distance from a SW to its closest and preceding sharp

27	Ratio of detected waveform types: SW to sharp

^*∗*^Basic features (wave-width, amplitude, kurtosis, amplitude to wave-width ratio, and skewness) were calculated from each detected waveform first, and their median/mean was taken as the features for the classification.

**Table 3:** List of parameters used for SVM: *C* denotes box constraint for soft margin, *σ* is a scaling factor for Gaussian kernel, *L* denotes tolerance with which the Karush-Kuhn-Tucker (KKT) conditions are satisfied, and *K* is the KKT violation level.

Target classification	Parameters	Value
EDs versus normal patterns	*C*	1
	*σ*	3
	*L*	0.01
	*K*	0

Sharps versus SWs	*C*	0.1
	*σ*	3
	*L*	0.05
	*K*	0

**Table 4:** Accuracy of classifying EDs and normal segments: PR denotes the proposed method, WL denotes the wavelet-based method, and PR + WL denotes a method in which the features of the proposed method and wavelet features were utilized together. Please note sensitivity of a type is equal to the specificity of the other type because this is binary classification.

Iteration number	Sens_ED_/Spec_Normal_			Sens_Normal_/Spec_ED_			Selectivity			Accuracy		
	PR	WL	PR + WL	PR	WL	PR + WL	PR	WL	PR + WL	PR	WL	PR + WL
1	95.00	94.44	87.22	93.85	89.53	95.80	93.87	89.63	95.64	94.42	91.99	91.51
2	93.92	93.92	90.61	93.73	89.24	95.65	93.73	89.33	95.55	93.83	91.58	93.13
3	91.71	92.82	85.64	93.67	89.78	95.75	93.63	89.84	95.55	92.69	91.30	90.69
4	91.67	94.44	90.00	93.36	88.91	95.49	93.33	89.02	95.38	92.51	91.68	92.74
5	93.92	95.58	90.06	93.13	89.18	95.23	93.15	89.31	95.13	93.53	92.38	92.64
6	96.11	93.89	89.44	93.35	89.41	95.74	93.40	89.50	95.61	94.73	91.65	92.59
7	91.11	92.78	87.22	93.23	89.44	95.47	93.19	89.51	95.32	92.17	91.11	91.35
8	95.03	88.95	88.40	93.86	89.86	95.87	93.88	89.84	95.72	94.44	89.41	92.13
9	95.03	91.16	87.29	93.46	89.45	95.51	93.49	89.49	95.35	94.24	90.31	91.40
10	96.67	92.78	86.67	93.41	89.43	96.13	93.48	89.50	95.95	95.04	91.11	91.40

Avg.	94.02	93.08	88.25	93.50	89.42	95.66	93.51	89.49	95.52	93.76	91.25	91.96
St dev.	1.94	1.90	1.69	0.26	0.28	0.25	0.26	0.25	0.23	1.00	0.86	0.79

**Table 5:** Classification accuracy between repeated sharps and SSW segments: the notations for the methods are the same as those used in Table 4.

Iteration number	Sens_sharp_/Spec_SSW_			Sens_SSW_ /Spec_Sharp_			Selectivity			Accuracy		
	PR	WL	PR + WL	PR	WL	PR + WL	PR	WL	PR + WL	PR	WL	PR + WL
1	100.00	0.00	50.00	69.54	97.70	91.95	70.56	94.44	90.56	84.77	48.85	70.98
2	100.00	85.71	100.00	74.14	86.21	90.23	75.14	86.19	90.61	87.07	85.96	95.11
3	83.33	33.33	66.67	74.86	90.29	90.29	75.14	88.40	89.50	79.10	61.81	78.48
4	100.00	83.33	100.00	65.52	95.98	84.48	66.67	95.56	85.00	82.76	89.66	92.24
5	85.71	28.57	42.86	75.29	99.43	95.40	75.69	96.69	93.37	80.50	64.00	69.13
6	83.33	83.33	66.67	75.29	87.93	86.78	75.56	87.78	86.11	79.31	85.63	76.72
7	83.33	66.67	83.33	74.71	93.68	89.08	75.00	92.78	88.89	79.02	80.17	86.21
8	50.00	66.67	50.00	76.00	86.29	94.29	75.14	85.64	92.82	63.00	76.48	72.14
9	28.57	57.14	42.86	77.01	87.93	87.93	75.14	86.74	86.19	52.79	72.54	65.39
10	83.33	66.67	100.00	77.59	93.10	90.23	77.78	92.22	90.56	80.46	79.89	95.11

Avg.	79.76	57.14	70.24	73.99	91.85	90.07	74.18	90.64	89.36	76.88	74.50	80.15
St dev.	23.15	28.10	23.97	3.69	4.83	3.29	3.18	4.16	2.83	10.62	12.84	11.22

**Table 6:** Classification accuracy for three types of iEEG segments: the notations are the same as those in Table 4.

Iteration number	Sens_Normal_			Spec_Normal_			Sens_Sharp_			Spec_Sharp_			Sens_SSW_			Spec_SSW_			Sel			Acc		
	PR	WL	PR + WL	PR	WL	PR + WL	PR	WL	PR + WL	PR	WL	PR + WL	PR	WL	PR + WL	PR	WL	PR + WL	PR	WL	PR + WL	PR	WL	PR + WL
1	93.72	88.79	96.04	95.00	95.00	86.67	100.00	0.00	50.00	95.69	98.22	99.05	68.97	93.68	79.89	97.62	90.52	96.86	93.26	88.82	95.71	91.83	77.70	84.75
2	93.79	89.31	95.76	93.92	93.37	91.16	85.71	57.14	100.00	96.09	96.00	98.92	74.14	82.76	82.18	97.40	93.17	96.70	93.42	89.17	95.51	90.18	85.29	94.12
3	93.69	90.05	95.65	92.82	93.92	86.19	83.33	16.67	66.67	96.28	98.00	99.02	72.00	86.29	77.14	97.08	91.89	96.47	93.27	89.93	95.28	89.20	79.47	86.86
4	93.39	89.08	95.46	92.22	94.44	91.11	100.00	33.33	100.00	95.82	97.61	98.80	65.52	92.53	77.59	97.16	91.45	96.44	92.88	89.11	95.13	90.68	83.07	93.23
5	93.02	89.03	95.14	93.92	95.58	90.06	57.14	28.57	14.29	96.12	99.02	99.38	73.56	95.40	87.36	96.57	89.99	95.66	92.63	89.10	94.93	85.06	82.93	80.31
6	93.27	89.50	95.59	95.56	93.33	91.11	83.33	50.00	66.67	96.66	97.00	99.14	74.14	82.18	80.46	96.26	92.32	96.25	92.91	89.34	95.29	89.87	84.06	88.20
7	93.10	89.50	95.23	91.67	92.22	89.44	66.67	33.33	66.67	96.00	97.59	98.78	72.99	86.78	80.46	96.81	91.82	96.29	92.71	89.41	94.94	86.20	81.87	87.81
8	93.94	89.90	95.57	95.58	90.06	88.40	50.00	33.33	50.00	96.51	96.99	99.14	76.00	79.43	84.00	97.09	92.73	96.34	93.57	89.67	95.33	84.85	80.41	85.58
9	93.58	89.58	95.52	94.48	90.61	88.40	14.29	42.86	28.57	96.83	97.00	99.04	74.71	80.46	77.59	96.37	92.41	96.25	93.17	89.38	95.13	78.38	82.15	80.89
10	93.39	89.55	96.01	96.67	94.44	87.78	83.33	33.33	66.67	96.36	97.93	98.95	77.59	89.66	80.46	96.73	91.53	96.93	93.09	89.52	95.70	90.68	82.74	87.80

Average	93.49	89.43	95.60	94.18	93.30	89.03	72.38	32.86	60.95	96.24	97.54	99.02	72.96	86.92	80.71	96.91	91.78	96.42	93.09	89.35	95.30	87.69	81.97	86.96
St dev.	0.30	0.39	0.29	1.60	1.83	1.84	26.26	16.14	27.17	0.36	0.83	0.18	3.48	5.72	3.17	0.44	0.97	0.36	0.31	0.32	0.28	4.11	2.24	4.49

**Table 7:** Distribution of outputs for each type of iEEG segment (S: repetitive sharp; SSW: runs of sharp-and-slow-wave; N: normal): the numbers of detected segments were summed over iterations and divided by the total number of corresponding target segments.

		PR			WL			PR + WL		
		N	S	SSW	N	S	SSW	N	S	SSW
Target	N	**93.49**	3.43	3.08	**89.43**	2.38	8.20	**95.60**	0.84	3.56
	S	7.94	**71.43**	20.63	25.40	**33.33**	41.27	12.70	**60.32**	26.98
	SSW	5.74	21.30	**72.96**	6.03	7.06	**86.91**	10.91	8.38	**80.71**

## Appendix

This appendix shows the detailed procedure to find local maxima from a given discrete signal.

Let *S* be a time-series signal and *s*_*i*_ a value of the *i*th data point of *S*.

(1) Set a checkpoint at *s*_2_ and a variable bUP to be false, where bUP is a Boolean variable to represent if the preceding signal from the checkpoint satisfies a specific condition.
(2) Test whether the checkpoint is a local maximum with the following steps. Let the data index of the check point be *c*.
  (A) Determine *s*_*c*_ as a local maximum and bUP as false if *s*_*c*_ > *s*_(*c*−1)_ and *s*_*c*_ > *s*_(*c*+1)_.
  (B) Set two variables bUP and *X* to be true and *c*, respectively, if *s*_*c*_ > *s*_(*c*−1)_ and *s*_*c*_ = *s*_(*c*+1)_.
  (C) Determine *s*_[(*c* + *X*)/2]_ and set bUP to be false if *s*_*c*_ = *s*_(*c*−1)_, *s*_*c*_ > *s*_(*c*+1)_ and bUp is true.
  (D) Set bUP to be false if *s*_*c*_ = *s*_(*c*−1)_ and *s*_*c*_ < *s*_(*c*+1)_.
(3) Move the checkpoint forward until it reaches the end of the signal.
